# Estrogen-dependent regulation of human uterine natural killer cells promotes vascular remodelling via secretion of CCL2

**DOI:** 10.1093/humrep/dev067

**Published:** 2015-03-27

**Authors:** D.A. Gibson, E. Greaves, H.O.D. Critchley, P.T.K. Saunders

**Affiliations:** 1 Medical ResearchCouncil Centre for Inflammation Research, The University of Edinburgh, The Queen's Medical Research Institute, 47 Little France Crescent, Edinburgh EH16 4TJ, UK; 2 Medical Research Council Centre for Reproductive Health, The University of Edinburgh, Queen's Medical Research Institute, 47 Little France Crescent, Edinburgh EH16 4TJ, UK

**Keywords:** uterine natural killer cell, estrogen receptor β, estradiol, angiogenesis, chemokine (C-C motif) ligand 2 CCL2

## Abstract

**STUDY QUESTION:**

Does intrauterine biosynthesis of estrogen play an important role in early pregnancy by altering the function of uterine natural killer (uNK) cells?

**SUMMARY ANSWER:**

Estrogens directly regulate the function of human uNK cells by increasing uNK cell migration and secretion of uNK cell-derived chemokine (C-C motif) ligand 2 (CCL2) that critically facilitates uNK-mediated angiogenesis.

**WHAT IS KNOWN ALREADY:**

uNK cells are a phenotypically distinct population of tissue-resident immune cells that regulate vascular remodelling within the endometrium and decidua. Recently we discovered that decidualisation of human endometrial stromal cells results in the generation of an estrogen-rich microenvironment in areas of decidualised endometrium. We hypothesize that intrauterine biosynthesis of estrogens plays an important role in early pregnancy by altering the function of uNK cells.

**STUDY DESIGN, SIZE, DURATION:**

This laboratory-based study used primary human uNK cells which were isolated from first trimester human decidua (*n* = 32).

**PARTICIPANTS/MATERIALS, SETTING, METHODS:**

Primary uNK cells were isolated from first trimester human decidua using magnetic cell sorting. The impact of estrogens on uNK cell function was assessed. Isolated uNK cells were treated with estrone (E1, 10^−8^ M) or estradiol (E2, 10^−8^ M) alone or in combination with the anti-estrogen ICI 182 780 (ICI, 10^−6^ M). uNK cell motility was assessed by transwell migration assay and time-lapse microscopy. Expression of chemokine receptors was assessed by quantitative PCR (qPCR) and immunohistochemistry, and angiogenic factors were assessed by qPCR and cytokine array. Concentrations of CCL2 in supernatants were measured by enzyme-linked immunosorbent assay. Angiogenesis was assessed in a human endometrial endothelial cell network formation assay.

**MAIN RESULTS AND THE ROLE OF CHANCE:**

Treatment with either E1 or E2 increased uNK cell migration (*P* = 0.0092 and *P* = 0.0063, respectively) compared with control. Co-administration of the anti-estrogen ICI blocked the effects of E1 and E2 on cell migration. Concentrations of C-X-C chemokine receptor type 4 (*CXCR4)* mRNA in uNK cells were increased by E2 treatment. The network formation assay revealed that conditioned media from uNK cells treated with E2 significantly increased human endometrial endothelial cell (HEEC) angiogenesis (*P* = 0.0029 versus control). Analysis of media from uNK cells treated with E2 using an antibody array identified CCL2 as the most abundant cytokine. Validation assays confirmed concentrations of CCL2 mRNA and protein were increased by E2 in uNK cells (*P* < 0.05 versus controls). Compared with the control, recombinant human CCL2 was found to increase HEEC network formation (*P* < 0.05) and neutralization of CCL2 in uNK conditioned media significantly decreased E2-dependent uNK-mediated network formation (*P* = 0.0006).

**LIMITATIONS, REASONS FOR CAUTION:**

Our results are based on *in vitro* responses of primary human cells and we cannot be certain that similar mechanisms occur *in vivo* in humans. Primary human uNK cells were isolated from first trimester decidua at a range of gestations (8–12 weeks), which may be a source of variation. Primary human uNK cells from non-pregnant endometrium were not assessed and therefore the responses of uNK cells to E2 treatment described in this study may be distinct to uNK cells from first trimester decidua.

**WIDER IMPLICATIONS OF THE FINDINGS:**

E2 is an essential regulator of reproductive competence. This study demonstrates a critical role for E2 in regulating cellular cross-talk within the endometrium during early pregnancy. We provide the first evidence that E2 directly regulates the function of human uNK cells by altering uNK cell migration and the secretion of uNK-derived angiogenic factors. We describe a novel mechanism of estrogen-dependent secretion of CCL2 which critically mediates uNK-dependent endometrial angiogenesis. Dysregulation of uNK cell function has been implicated in the aetiology of early implantation disorders and disorders of pregnancy. These novel findings provide unique insight into the regulation of uNK cell activity during the establishment of pregnancy in women and highlight key processes which may be targeted in future therapeutic strategies.

**STUDY FUNDING/COMPETING INTEREST(S):**

Studies undertaken in the authors' laboratory were supported by MRC Programme Grant G1100356/1 to P.T.K.S. The authors have no conflicts of interest to disclose.

## Introduction

The endometrium is a complex multicellular tissue that undergoes dynamic remodelling in order to establish a microenvironment capable of supporting a pregnancy. During the establishment of pregnancy endometrial remodelling is characterized by three key processes; influx of uterine natural killer (uNK) cells, decidualisation (differentiation) of stromal fibroblasts and remodelling of the endometrial vasculature. Coordinated regulation of these processes is critical for pregnancy success.

uNK cells are a phenotypically distinct population of tissue-resident immune cells that are abundant in secretory phase endometrium and first trimester decidua (King *et al.*, 1991). uNK cell numbers increase from Day 22 of the standardized 28 day cycle (Russell *et al.*, 2013), coincident with increasing levels of progesterone, increasing to be become the predominant leukocyte population in first trimester decidua. The mechanisms that control accumulation and localization of uNK cells in the uterus remain unresolved although there is evidence to suggest that *in situ* proliferation, and recruitment and differentiation of NK cell precursors and/or haematopoietic stem cells may contribute to the rapid increase in cell numbers in the endometrium during the establishment of pregnancy (King *et al.*, 1991; Jones *et al.*, 2004; Sentman *et al.*, 2004; Kitaya *et al.*, 2005, 2007; Wu *et al.*, 2005; Carlino *et al.*, 2008; Kane *et al.*, 2009; Vacca *et al.*, 2011). Although uNK cells do not express progesterone receptors (Henderson *et al.*, 2003), progesterone indirectly promotes accumulation and differentiation of uNK cells in the endometrium. For example, incubation with progesterone increases synthesis and secretion of interleukin (IL)-15 from human endometrial stromal cells *in vitro* (Okada *et al.*, 2000) and treatment of women with the selective progesterone receptor modulator Asoprisnil results in a striking reduction in both *IL15* mRNA and the number of CD56+ uNK cells detected in non-pregnant endometrium (Wilkens *et al.*, 2013).

During the mid-secretory phase, decidualisation (differentiation) of the stromal cells is first detected adjacent to the spiral arteries in the upper (functional) layer of the endometrium in response to the rising levels of ovarian-derived progesterone. Decidualisation then spreads ‘wave-like’ throughout the tissue and if pregnancy ensues the cells remain *in situ* within the decidua of early pregnancy (reviewed in (Gellersen and Brosens, 2014)). Notably, decidualisation stimulates endometrial stromal cells to secrete a number of growth factors and cytokines that are key regulators of immune cell function and vascular development during endometrial remodelling. Recently we discovered that decidualisation of human endometrial stromal cells also results in biosynthesis of estrogens which we believe may be important in regulating early pregnancy tissue remodelling (Gibson *et al.*, 2013). We described expression of the estrogen biosynthetic enzyme aromatase in both first trimester human decidua and in decidualised human endometrial stromal cells. In *in vitro* assays we detected secretion of significant (nM) concentrations of both estrone (E1) and estradiol (E2), from decidualised stromal cells (Gibson *et al.*, 2013). Given that human uNK cells are immunopositive for estrogen receptor (ER)β in tissue sections (Henderson *et al.*, 2003), these new data prompted us to explore whether the uNK cell population could be responding to local concentrations of bio-available estrogens in decidualised endometrium in early pregnancy.

To date there is only limited evidence to suggest that uNK cells may be directly regulated by estrogens. DeLoia *et al.* reported that in women who had received an artificial hormonal regimen to mimic the secretory phase (progesterone and E2), increased numbers of uNK cells were detected in endometrial biopsies when circulating estrogen concentrations were increased (DeLoia *et al.*, 2002) but whether the effects of estrogens on uNK cells were direct or indirect was not explored. Most of the data generated to explore the function of uNK cells in early pregnancy events has relied upon studies in mouse models including recent reports that uNK cells play a role in regulating uterine vascular remodelling in early pregnancy (Croy *et al.*, 2012; Hofmann *et al.*, 2014) and reviewed in (Lima *et al.*, 2014). However, as the uNK cells in mouse implantation sites are reported to be devoid of ER mRNAs (Borzychowski *et al.*, 2003), we are only able to gain a greater understanding of uNK cell regulation by estrogens and their potential regulatory role in human pregnancy by performing studies using primary human uNK cells.

Isolated human uNK cells are reported to secrete angiogenic factors such as vascular endothelial growth factor (VEGF) and placental growth factor (PLGF) which promote angiogenesis *in vitro* and *in vivo* (Hanna *et al.*, 2006; Wallace *et al.*, 2012). In women, histological studies have revealed that uNK cells accumulate around spiral arterioles and are abundant in perivascular sites in both late secretory phase endometrium and in early pregnancy decidua (Bulmer *et al.*, 1991). There is convincing evidence that uNK cells regulate vascular remodelling during trophoblast invasion and placentation (Lash *et al.*, 2010, 2011; Wallace *et al.*, 2012). However, there is also accumulating evidence that in early pregnancy spiral artery modifications in the decidua can occur independently of cellular interactions with trophoblast cells (Craven *et al.*, 1998). For example, Histological studies have demonstrated that changes in spiral arteries, such as loss of vascular smooth muscle cells and endothelial cell breaks, are associated with maternal uNK cells but not fetal extravillous trophoblasts (Craven *et al.*, 1998; Smith *et al.*, 2009). Studies utilizing co-cultures of placental villi and decidual explants have demonstrated that decidual macrophages and uNK cells initiate early stages of vascular remodelling (Hazan *et al.*, 2010). In addition, recent data support a role for uNK cells in promoting the early stages of endometrial vascular remodelling through secretion of angiogenic growth factors such as angiopoietin-2 (ANGPT2) (Robson *et al.*, 2012). Gestational differences in the secretion of angiogenic growth factors from uNK cells purified and maintained for 48 h in culture have been reported. For example, uNK cells recovered at 8–10 weeks gestation secreted more of the angiogenic growth factors VEGF-C and ANGPT2 than uNK cells recovered at 12–14 weeks gestation (Lash *et al.*, 2006). Taken together these data suggest human uNK cells play a key role in directly regulating vascular remodelling during early pregnancy.

Disorders of pregnancy are often associated with impaired vascular remodelling and some studies have suggested that this may be associated with alterations in uNK cell number and function although the data are conflicting. For example, pre-eclampsia (PE) is a complex syndrome with a multifactorial aetiology characterized by vascular pathology and associated with defects in uterine spiral arteries. The associated role of uNK cells in spiral artery modifications has led researchers to speculate that uNK cells may be dysregulated in PE although this is still to be fully elucidated. Although some studies report that *decreased* numbers of uNK cells are associated with PE and fetal growth restriction (Williams *et al.*, 2009; Lockwood *et al.*, 2013), Bachmayer *et al.* reported *increased* uNK cells in term placental tissues from women with PE (Bachmayer *et al.*, 2006). uNK cell density is also reported to be proportional to endometrial angiogenesis in women with recurrent reproductive failure (Quenby *et al.*, 2009). It has been proposed that an increase in the number of uNK cells in the stroma underlying the luminal epithelium of the endometrium of women during the secretory phase can be used as a diagnostic test to guide therapies for women suffering from infertility (Tang *et al.*, 2013). However, a recent meta-analysis that considered 22 studies found no significant difference in the percentage of uNK cells between fertile and infertile women and concluded that NK cell analysis or immune cell therapy should only be considered in the context of clinical research until further studies were conducted (Seshadri and Sunkara, 2014).

In conclusion, it is generally accepted that coordinated cellular cross-talk between decidualised stromal cells, uNK cells and the endometrial vasculature is critical for appropriate regulation of endometrial remodelling in early pregnancy. Despite an association between dysregulation of uNK function and disorders of pregnancy, the fundamental mechanisms underlying normal regulation of human uNK cell function remain unresolved. In the present study we tested the hypothesis that estrogens produced by decidualised stromal cells (Gibson *et al.*, 2013) can have a *direct* impact on the function of uNK cells. We assessed the impact of estrogens on human uNK cell motility and investigated whether E2 could modulate changes in vascular function by directly inducing changes in production of secreted factors from uNK cells.

## Materials and Methods

### Isolation of uNK cells

Decidual samples (*n* = 32) were obtained from women requesting surgical termination of pregnancy procedure with a mean gestation of 10 weeks (range 8–12 weeks, Supplementary Table SI) dated according to the woman's reported last menstrual period. All women had an ultrasound scan to confirm viability of pregnancy and gestational age; none were over 12 weeks gestation. Local ethical committee approval was granted and written informed patient consent was obtained prior to tissue collection by a dedicated research nurse. (Ethical approval held by HODC; LREC/05/51104/12 and LREC/10/51402/59). Primary human uNK cells were isolated from human first trimester decidua as described previously (Kane *et al.*, 2009) using magnetic separation and the MACS^®^ system (Miltenyi Biotech, Germany). uNK cells were isolated following CD3 depletion and CD56 positive selection using the appropriate antibody-coated magnetic microbeads. Isolated uNK cells were immuno-phenotyped using flow cytometry (Supplementary Fig. S1 and Supplementary Table SII).

### uNK cell migration assay

Migration of uNK cells was assessed using a modified transwell migration assay. Isolated uNK cells were treated with vehicle control (dimethyl sulfoxide, DMSO) or either 10^−8^ M E1 or 10^−8^ M E2, alone or in combination with 10^−6^ M ICI 182 780 (ICI) for 1 h prior to assay (*n* = 6 per group). Following treatment, cells were pelleted and resuspended in culture media (Phenol red-free RPMI 1640 plus 10% charcoal stripped fetal calf serum, Invitrogen) prior to migration assay. Cell culture plates were coated with a 1.6 mg/ml collagen gel (GIBCO/Invitrogen, A10644-01); serum-free, Phenol red-free RPMI 1640 media was added to the well on top of the collagen matrix. Cell culture plates were collagen coated prior to assay to provide an adherent surface for attachment of migrated uNK cells. Cell culture inserts with a 5 µm membrane pore (Costar/Corning, 3421, NY, USA) were then placed into the well into which the uNK suspension was added (2 × 10^5^ cells per insert). Cells were treated in duplicate and left in the incubator to migrate for 1 h. Migrated cells were counted by imaging 8 random fields using Axiovert 200 Inverted Fluorescent Microscope (Zeiss) and the mean number of migrated cells was calculated for each treatment. The fold change compared with the vehicle control was plotted.

### Immunofluorescence

Expression of CD56, CXCR4 and C-X-C motif chemokine 12 (CXCL12) in sections of human decidua (chosen because they have a very high abundance of CD56+ cells) was determined by immunohistochemistry using standard protocols and tyramide signal amplification detection system (Perkin Elmer). Primary antibodies; CXCL12 (mouse monoclonal antibody; R&D systems MAB350), CXCR4 (mouse monoclonal antibody; R&D systems MAB172) and CD56 (mouse monoclonal antibody; Zymed 18-0152) were incubated overnight at 4°C. Fluorescent images were examined using Zeiss LSM 510 Meta-Confocal microscope.

### uNK cell chemokinesis

The chemokinesis of uNK cells was assessed using Ibidi µ-Slide Chemotaxis^3D^ chamber slides (Ibidi, 80 326, supplied by Thistle Scientific Ltd, Uddingston, UK). Isolated uNK cells were suspended in a collagen matrix (as above) and the response to E2 treatment was measured using time-lapse microscopy. Chamber slides were set up containing serum-free phenol red-free RPMI 1640 media in both reservoirs and uNK cells were treated ±E2. Cells were imaged every 5 min for 4 h (*n* = 30 per treatment) using an Axiovert 200 Inverted Fluorescent Microscope (Zeiss). Data were analysed using ImageJ (manual cell tracking plug-in) and chemotaxis and migration tool software (Ibidi).

### Network formation assay

The formation of networks by telomerase immortalized human endometrial endothelial cells (HEEC, gifted from Yale University, New Haven, CT, USA; (Schatz *et al.*, 2000)) was assessed as described previously (Greaves *et al.*, 2013). HEECs were derived from primary human endometrial endothelial cells as described in Schatz *et al.* (2000) and immortalized as described in Krikun *et al.* (2005). HEEC express classic endothelial cell markers, such as von Willebrand factor (VwF) and platelet/endothelial cell adhesion molecule 1 (PECAM1, CD31), and transcriptional analysis by microarray confirmed 93% identity with parent primary endothelial cells (Krikun *et al.*, 2005); prior to use in the assays described in this paper expression of CD31 and VwF was reconfirmed as described previously (Greaves *et al.*, 2013).

Isolated uNK cells were cultured at a density of 1 × 10^6^ cells per/ml and treated with 10^−8^ M E2, 10^−6^ M ICI or a combination of E2 and ICI for 24 h (*n* = 10 per group). Following treatment, cells were pelleted and the supernatant, uNK cell conditioned media (CM), were stored at −80°C until assay. uNK CM was thawed immediately prior to assay and warmed to 37°C. uNK CM or phenol red-free RPMI 1640 media containing recombinant human chemokine (C-C motif) ligand 2 (CCL2) (RnD; Cat #279-MC-010) was added to the bottom chambers. Where appropriate, the pure anti-estrogen ICI was added into the upper chamber for 1 h before the addition of uNK-CM. Neutralization of CCL2 was achieved by incubating treatment media with a monoclonal antibody to CCL2 (mAb-CCL2; Monoclonal Mouse IgG1 Clone # 24822, RnD; Cat # MAB279) for 1 h prior to assay. HEEC were incubated for 16 h at 37°C to allow formation of networks. Network formation was visualized using an Axiovert 200 Inverted Fluorescent Microscope and analysed as described previously (Greaves *et al.*, 2013). Network formation was quantified as fold change relative to comparator.

### Angiogenesis RT2 profiler PCR array

RT2 profiler PCR arrays (SABiosciences, Frederick, MD, USA) were utilized to analyse a targeted panel of 84 genes related to angiogenesis. uNK cells were isolated from three patients and treated ±E2 for 2 h. Total RNA was extracted from uNK cells using a Qiagen RNAeasy kit (Qiagen, UK) according to manufacturer's instructions and retrotranscribed using a RT2 single strand cDNA synthesis kit (SAB Biosciences, Frederick, MD, USA).

PCR array data analysis was performed using the SABiosciences web portal according to the ^ΔΔ^Ct method (http://www.SABiosciences.com/pcrarrayanalysis.php).

Expression of mRNAs encoding C-X-C chemokine receptor type 4 (*CXCR4)*, Interleukin-15 receptor alpha (*IL15RA)*, interferon gamma (*IFNG**)*** and *CCL2* was assessed by quantitative PCR (qPCR) according to standard protocols. Samples were quantified using the comparative ΔΔCt method with ribosomal 18S as internal control reference gene. The primers/probes are given in Supplementary Table SIII. PCR primers were designed using the Roche Universal Probe Library Assay Design Center. Primer efficiency was between 90 and 110% and amplicon sizes were confirmed (Supplementary Fig. S2).

### Cytokine array

The relative abundance of 60 cytokines in uNK CM was assessed using Raybiotech Human Cytokine Array (Cat# AAH Cyt-6) and visualized using the Odyssey^®^ system (LiCor). Relative cytokine expression levels were determined by assessing signal intensities using Odyssey application software.

### Milliplex assay

The concentration of CCL2 in uNK CM (*n* = 5 per group) was quantified using a customized singleplex bead enzyme-linked immunosorbent assay (HCYTOMAG-60 K, Millipore, Schwalbach, Germany) according to manufacturer's instructions. Standards and samples were measured in duplicate using a Bio-Plex 200 HTF machine and analysed using BioPlex Manager Software (Version 5, BioRad).

### Statistical analysis

Statistical analysis was performed using Graphpad prism (GraphPad Software, Inc., La Jolla CA, USA). Student's *t*-test was used to determine significance between treatments in data that were normally distributed. Non-parametric testing was utilized where sample sizes were insufficient to confirm normality of data distribution. Mann–Whitney test was used to assess differences between treatments. Where data were analysed as fold change, significance was tested using one-sample *t*-test and a theoretical mean of 1. Criterion for significance was *P* < 0.05. All data are presented as mean ± SEM.

## Results

### Estrogens promote uNK cell motility

**Figure 1:**
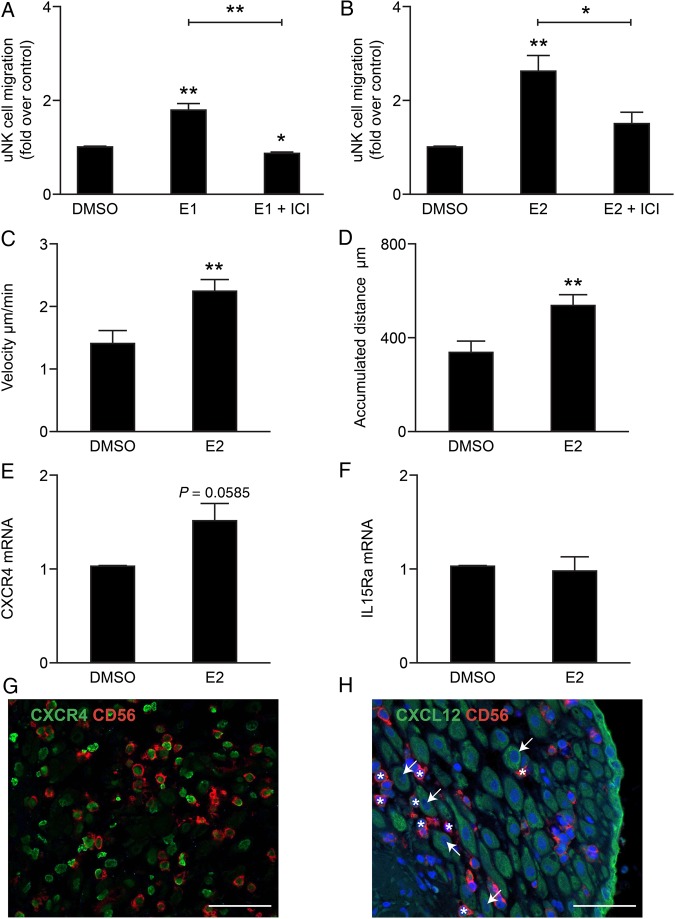
Estrogens promote uNK cell migration and chemokinesis. Uterine natural killer (uNK) cells were treated in duplicate with estrone (E1) or estradiol (E2) alone or in combination with the anti-estrogen ICI 182, 780 (ICI). Relative fold change responses were compared with vehicle control (dimethyl sulfoxide, DMSO). (**A**) E1 significantly increased uNK cell migration by ∼1.8-fold (*n* = 6, One-sample *t*-test, *P* = 0.0092), co-administration of ICI blocked E1-mediated increased migration (Mann–Whitney test, *P* = 0.0022). (**B**) E2 significantly increased uNK cell migration 2.6-fold (*n* = 6, One-sample *t*-test, *P* = 0.0063), co-administration of ICI blocked E2-mediated increased migration (Mann–Whitney test, *P* = 0.0260). uNK cell motility in cells treated with vehicle control (DMSO, *n* = 30 cells) or E2 (*n* = 30 cells) was assessed using time-lapse video microscopy (Supplementary Videos S1 and S2). (**C**) E2 treatment significantly increased uNK cell velocity (µm/min) compared with vehicle control (Student's *t*-test, *P* = 0.0016). (**D**) E2 treatment significantly increased the accumulated uNK cell distance (µm) compared with vehicle control (Student's *t*-test, *P* = 0.0016). The expression of chemokine receptors in uNK cells in response to E2 was assessed by quantitative PCR (qPCR); fold change compared with vehicle control was determined. (**E**) E2 tended to increase concentrations of mRNAs encoding C-X-C chemokine receptor type 4 (*CXCR4)* (*n* = 5, One-sample *t*-test, *P* = 0.0585). (**F**) *IL15RA* mRNA was unchanged. The expression of the uNK chemoattractant C-X-C motif chemokine 12 (CXCL12) and its receptor CXCR4 were assessed in first trimester human decidua by immunohistochemistry. (**G**) Positive staining for CXCR4 (green) was detected in CD56-positive uNK cells (red staining). (**H**) CXCL12 did not co-localize with CD56-positive uNK cells (asterisks); however, positive staining was detected in decidualised stromal cells (arrows) and epithelial cells (green staining) in decidua. Nuclear counterstain DAPI (blue staining). Scale bar 50 µm. *P < 0.05, **P < 0.01.

In agreement with results of tissue immunohistochemistry (Henderson *et al.*, 2003) uNK cells freshly isolated from first trimester decidua (determined to be >93% pure; Supplementary Fig. S1) were shown to express ERβ mRNA and protein but were immunonegative for ERα (Supplementary Fig. S3). To determine whether estrogens could have a direct impact on uNK cell motility, cells were treated with either E1 (10^−8^ M) or E2 (10^−8^ M) in the presence or absence of the anti-estrogen ICI (fulvestrant, 10^−6^ M). Treatment with E1 or E2 significantly increased uNK cell migration (Fig. 1A and B; *n* = 6 per treatment, *P* = 0.0092 and *P* = 0.0063, respectively) and co-administration of ICI blocked the effect of either E1 (*P* = 0.0022) or E2 (*P* = 0.026).

To complement and extend these studies, uNK cell motility was recorded using live cell imaging and time-lapse microscopy (Supplementary Videos S1 and S2). When cell movement was analysed using the chemotaxis and migration tool software (Ibidi) an increase in cellular activity was readily apparent following exposure to E2. Chemokinesis (non-directed cell movement) was measured: E2 treatment (10^−8^ M) was found to significantly increase both the velocity and the accumulated distance travelled by uNK cells (Fig. 1C and D, *P* < 0.01, *n* = 30 per treatment) compared with control (DMSO).

During decidualisation endometrial stromal cells secrete estrogens (Gibson *et al.*, 2013) as well as uNK chemoattractants such as CXCL12 and IL-15 (Kitaya *et al.*, 2005; Wu *et al.*, 2005). To investigate whether E2 could increase responsiveness of uNK cells to chemoattractants, the expression of the CXCL12 receptor (CXCR4) and the IL-15 receptor alpha (IL15Ra) was assessed in uNK cells by qPCR. E2 treatment up-regulated concentrations of mRNAs encoding *CXCR4* (Fig. 1E, *P* = 0.0585, *n* = 5) while concentrations of *IL15RA* were unchanged by E2 (Fig. 1F). Consistent with previous flow cytometry studies (Wu *et al.*, 2005), fluorescent immunohistochemistry analysis of human decidua revealed that CD56+ uNK cells were CXCR4 positive (Fig. 1G). Furthermore, uNK cells (asterisks) appeared to accumulate near to CXCL12-positive decidualised stromal cells (white arrows, Fig. 1H). Interestingly, CXCR4 was immunolocalised to the nuclei of CD56+ cells, consistent with nuclear re-localization as a result of CXCL12 stimulation (Wang *et al.*, 2009).

### Estradiol promotes uNK-mediated angiogenesis

**Figure 2:**
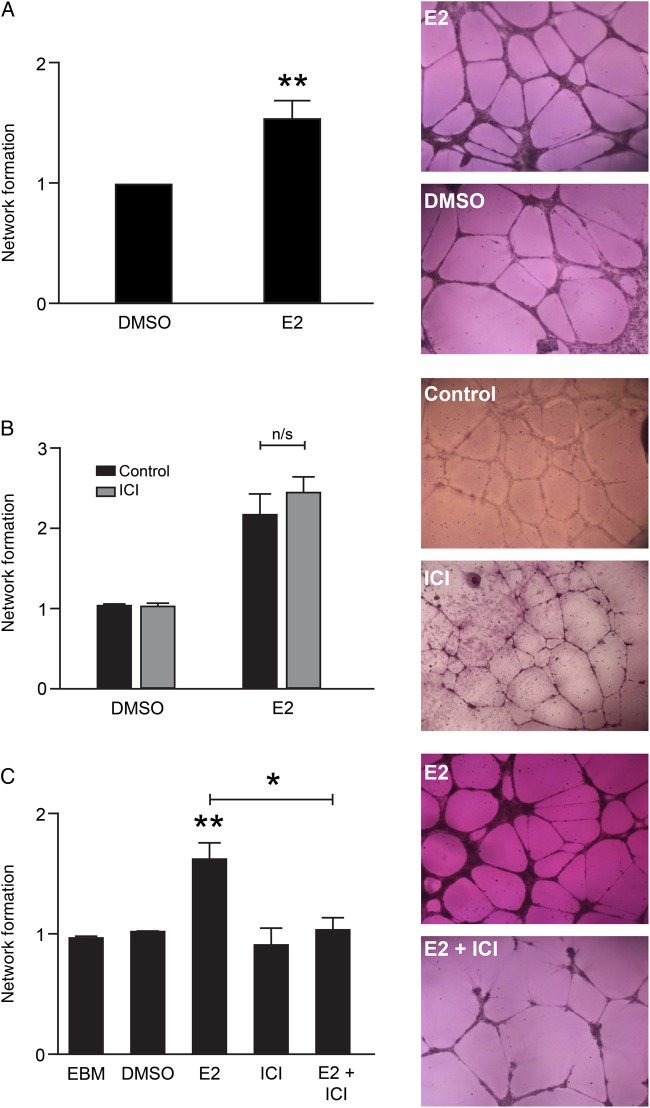
Estradiol promotes uNK-mediated network formation in endometrial endothelial cells via uNK estrogen receptor. Human endometrial endothelial cell (HEEC) network formation was assessed in response to uNK conditioned media (CM). Network formation was quantified as fold change relative to vehicle control. (**A**) CM from uNK cells treated with E2 significantly increased network formation in HEECs (*n* = 10, One-sample *t*-test, *P* = 0.0029). (**B**) Blocking of HEEC estrogen receptor (ER)β with ICI did not affect uNK-mediated increased network formation (n/s: not significant, *n* = 3, Mann–Whitney test). (**C**) CM from uNK cells treated with ICI or uNK cells treated with E2 in combination with ICI (E2+ICI) had no significant impact on network formation. CM from uNK cells treated with E2 significantly increased network formation relative to all treatments including endothelial basal growth media (EBM, *P* = 0.002). CM from uNK cells co-treated with E2 and ICI significantly decreased network formation compared with CM from uNK cells treated with E2 alone (*n* = 7, Mann–Whitney test, *P* = 0.0136). *P < 0.05, **P < 0.01. Images were captured at ×5 magnification using the Axiovert 200 Inverted Fluorescent Microscope.

uNK cells are reported to secrete angiogenic factors and to promote angiogenesis *in vitro* and *in vivo* (Hanna *et al.*, 2006). Recent reports suggest uterine artery remodelling *and* angiogenesis both occur in early pregnancy (Lima *et al.*, 2014). We investigated the effect of E2 on uNK-mediated angiogenesis using an established *in vitro* assay (Greaves *et al.*, 2013) of endothelial network formation which is considered a morphometric assay that models the reorganization stage of angiogenesis. HEEC network formation was assessed in response to CM from uNK cells treated with E2 (10^−8^ M), the anti-estrogen ICI (10^−6^ M) or E2 in combination with ICI. CM from uNK cells treated with E2 significantly increased network formation compared with control (Fig. 2A, *P* = 0.0029, *n* = 10). We have previously reported that HEEC can respond to estrogens via activation of ERβ (Greaves *et al.*, 2013); therefore we blocked any indirect stimulation of HEEC by E2 present in CM by pre-treating HEEC with the anti-estrogen ICI (10^−6^ M). Pretreatment with ICI did not affect HEEC network formation in response to uNK CM (Fig. 2B, *n* = 3). Finally, we confirmed that the secretion of pro-angiogenic factors from uNK cells in response to E2 was receptor mediated, as CM from uNK cells treated with E2+ICI did not increase HEEC network formation (Fig. 2C, *P* = 0.0136, *n* = 7).

### Targeted gene analysis reveals regulation of cytokines in uNK cells

**Figure 3:**
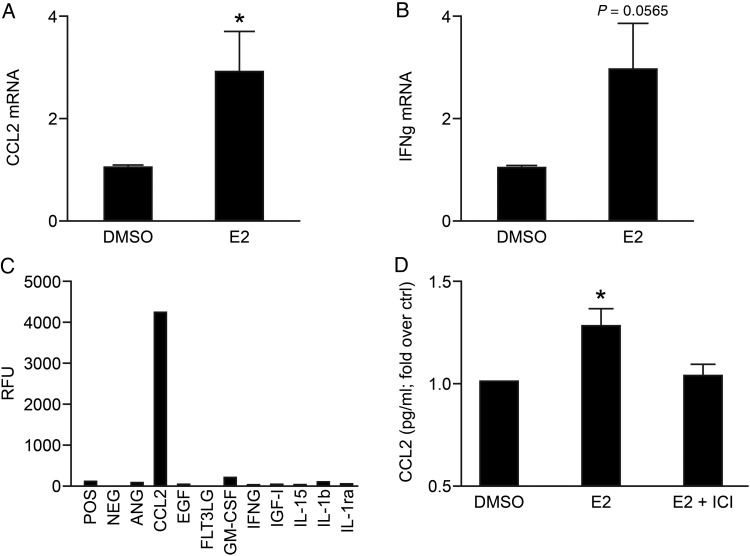
E2-dependent regulation of angiogenic factors in uNK cells. The top two targets identified by PCR array; chemokine (C-C motif) ligand 2 (CCL2) and interferon gamma (IFNγ), were selected for follow-up validation by qPCR. Fold change compared with vehicle control was determined. (**A**) E2 significantly increased expression of *CCL2* mRNA (*n* = 10, One-sample *t*-test, *P* = 0.0394) compared with control (DMSO). (**B**) Concentrations of mRNAs encoding *IFNG* tended to be increased by E2 treatment (*P* = 0.0565, *n* = 9). (**C**) Cytokines/growth factors in CM from uNK cells treated with E2 were assessed by cytokine array; levels of IFNγ were low/undetectable, CCL2 was abundant. (**D**) Concentrations of CCL2 were assessed by Milliplex bead assay. Concentrations of CCL2 were significantly increased in CM from uNK cells treated with E2 (*n* = 5, One-sample *t*-test, *P* = 0.0408 versus control). *P < 0.05, **P < 0.01.

To investigate whether treatment with E2 had an impact on expression of genes known to play a role in angiogenesis, mRNAs expressed by uNK cells were assessed using a targeted PCR array (SAB biosciences). The identity of mRNAs with altered expression is summarized in Table I. Messenger RNAs encoded by *VEGFC* and *PLGF,* previously reported to be uNK-derived angiogenic factors (Hanna *et al.*, 2006), were not detected and *VEGFA* expression was not affected by E2 treatment (Table I, *n* = 3). *CCL2* and *IFNG* were selected for follow-up validation as these were the top two targets that exhibited greatest increased regulation in response to E2. Targeted analyses of *IFNG* and *CCL2* were validated in a separate larger sample set of E2-treated uNK cells using qPCR and were increased in response to 10^−8^ M E2 (Fig. 3A; *P* = 0.0394, *n* = 10 and Fig. 3B; *P* = 0.0565, *n* = 9, respectively).

**Table I:** Differential expression of angiogenesis genes in uterine natural killer (uNK) cells treated with estradiol (E2).

Gene of interest	Fold change	*P*-value
*VEGFA*	1.007	0.756185
*MMP9*	1.4272	0.150482
*PECAM1*	1.7144	0.29493
*MMP2*	1.7916	0.066499
*CXCL10*	1.9562	0.446108
*TIMP1*	2.6924	0.105164
***CCL2***	**2.8044**	**0.128538**
***IFNG***	**4.4089**	**0.108815**

Top targets that were subsequently validated are listed in bold.

uNK cells were treated with control (dimethyl sulfoxide) or E2 for 2 h (*n* = 3) and the differential expression of angiogenesis genes was determined. PCR array data analysis was performed using the SABiosciences web portal according to the ^ΔΔ^Ct method (http://www.SABiosciences.com/pcrarrayanalysis.php) and gene expression expressed as fold change relative to vehicle control. The *P*-values are calculated based on a Student's *t*-test of the values for each gene in the control group and treatment groups. Angiogenesis genes that were differentially up-regulated in response to E2 are shown. Expression of vascular endothelial growth factor A (*VEGFA*) is also shown for comparison.

MMP9, matrix metallopeptidase 9; PECAM1, platelet/endothelial cell adhesion molecule 1; MMP2, matrix metallopeptidase 2; CXCL10, chemokine (C-X-C motif) ligand 10; TIMP1, TIMP metallopeptidase inhibitor 1; CCL2, chemokine (C-C motif) ligand 2; IFNG, interferon gamma.

To assess if changes in gene transcription were reflected in alterations in secretion of pro-angiogenic factors the relative abundance of cytokines/growth factors in uNK-E2 CM was assessed using a Human Cytokine Array. Levels of IFNG were low but CCL2 was abundant in uNK-E2 CM compared with other cytokines/growth factors (Fig. 3C). The concentration of CCL2 was quantified by Milliplex bead assay. Concentrations of CCL2 (mean concentration 0.1 ng/ml) were significantly increased in CM from E2-treated uNK cells (Fig 3D, *P* = 0.0408, *n* = 5).

### E2-dependent secretion of CCL2 promotes uNK-mediated angiogenesis

**Figure 4:**
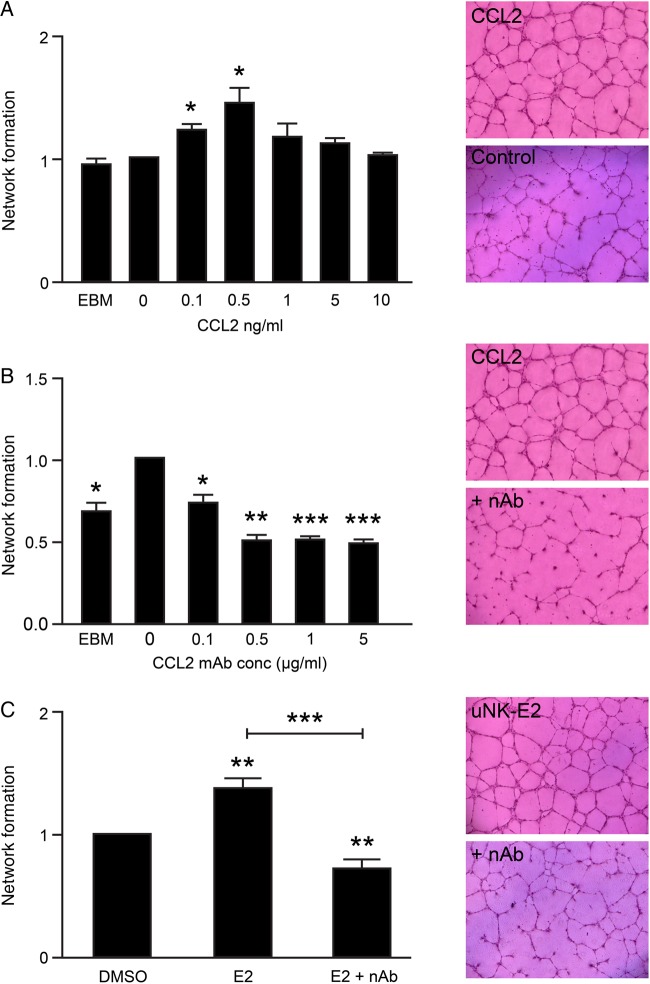
E2-dependent secretion of CCL2 regulates uNK-mediated angiogenesis. HEEC network formation was assessed in response to recombinant human CCL2 (rCCL2). (**A**) Network formation was significantly increased in response to 0.1 ng/ml rCCL2 (*n* = 4, One-sample *t*-test, *P* = 0.0348) and maximal in response to 0.5 ng/ml (*n* = 4, One-sample *t*-test, *P* = 0.047). (**B**) HEEC were incubated with 0.5 ng/ml rCCL2 and increasing concentrations of a monoclonal antibody to CCL2 (mAb-CCL2). HEEC network formation was assessed relative to 0 µg/ml mAb-CCL2. Neutralization of rCCL2 with mAb-CCL2 significantly inhibited HEEC network formation at concentrations of 0.1 µg/ml (*P* = 0.0174) and greater (*n* = 4, One-sample *t*-test, 0.5 µg/ml; *P* = 0.0026, 1 and 5 µg/ml; *P* = 0.0005). (**C**) uNK-E2 CM was incubated with 0.5 µg/ml mAb-CCL2 and HEEC network formation was assessed relative to control uNK CM (DMSO). Neutralization of CCL2 significantly reduced HEEC network formation (*n* = 8, One-sample *t*-test, *P* = 0.0069) and reversed uNK-E2-mediated increased network formation (*n* = 8, Mann–Whitney, *P* = 0.0006). *P < 0.05, **P < 0.01, ***P < 0.001. Images were captured at ×5 magnification using the Axiovert 200 Inverted Fluorescent Microscope.

To investigate whether CCL2 could directly promote endometrial angiogenesis, the impact of recombinant human CCL2 (rCCL2) on network formation by HEEC was assessed. A biphasic dose-dependent increase in HEEC network formation was detected in response to rCCL2 that was significant at 0.1 ng/ml (Fig. 4A, *P* = 0.0348, *n* = 4) and 0.5 ng/ml (Fig 4A, *P* = 0.0470, *n* = 4) but inhibited at concentrations ≥5 ng/ml. Interestingly, the concentration of CCL2 detected in uNK CM (0.1 ng/ml) was comparable to the dose of rCCL2 which produced a significant response. The capacity for increasing concentrations of a monoclonal antibody to CCL2 (mAb-CCL2) to neutralize rCCL2-mediated network formation was tested against 0.5 ng/ml rCCL2. Incubation with mAb-CCL2 for 1 h prior to assay was sufficient to significantly inhibit network formation at mAb-CCL2 concentrations of 0.1 µg/ml (*P* = 0.0174, *n* = 4) and greater (Fig. 4B, 0.5 µg/ml; *P* = 0.0026, 1 and 5 µg/ml; *P* = 0.0005, *n* = 4).

To confirm whether CCL2 was the key E2-dependent factor secreted by uNK cells that could induce changes in vascular endothelial cells, CM from uNK cells treated with E2 was incubated with 0.5 µg/ml mAb-CCL2 before it was used to assess HEEC network formation. Neutralization of CCL2 resulted in a significant decrease in E2-dependent uNK-mediated network formation (Fig. 4C, *P* = 0.0006, *n* = 8).

## Discussion

This study demonstrates that estrogens directly regulate the bioactivity of human uNK cells. Although there has been evidence to suggest uNK cells can be regulated indirectly by sex steroid hormones such as progesterone, this study provides the first evidence that estrogens are direct mediators of uNK cell function. Collectively, our novel findings indicate that estrogens increase uNK cell migration and responsiveness to chemoattractants. We report evidence that secretion of CCL2 from uNK cells is ER-dependent and that CCL2 critically facilitates uNK-mediated endometrial angiogenesis. These results support our hypothesis that estrogens synthesized within endometrial tissue promote regulation of uNK cells, leading to ER-dependent secretion of pro-angiogenic factors that promote vascular remodelling.

**Figure 5:**
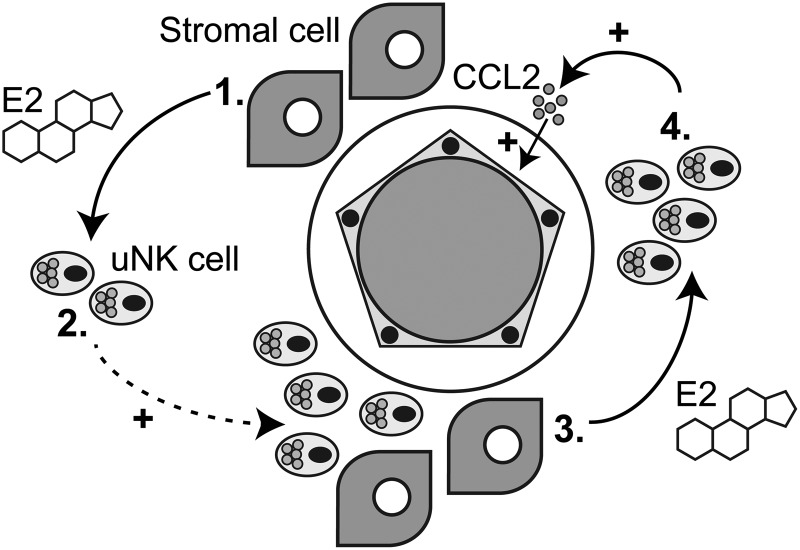
Summary: Regulation of endometrial function in early pregnancy. Stromal cells and uNK cells interact with endometrial arterioles to promote vascular remodelling. **1**. In the decidua, stromal cells secrete estrogens such as E1 and E2. **2**. E2 increases uNK cell motility and migration both directly and by increasing the expression of the chemokine receptor CXCR4. This action of E2 may promote localization/recruitment of uNK cells in the perivascular niche. **3**. E2 acts directly on uNK cells to promote secretion of pro-angiogenic factors including CCL2. **4**. ER-dependent secretion of CCL2 promotes endothelial cell angiogenesis and modulation of vascular function.

Understanding the dynamic changes that take place in the endometrium during the establishment of pregnancy is challenging. Our results provide new insights into the regulation of human endometrial remodelling and highlight the importance of cellular cross-talk between decidualised stromal cells, uNK cells and the endometrial vasculature. Decidualisation of endometrial stromal cells is a progressive process characterized by a time-dependent change in the ratios of secreted estrogens (E1 and E2). We have previously demonstrated that primary endometrial stromal cells induced to decidualise *in vitro* initially secrete a high E1:E2 ratio, followed by a progressive increase in E2 such that the concentration of E1 is equivalent to that of E2 when cells are fully transformed (Gibson *et al.*, 2013). Interestingly, in the present study, similar molar concentrations of E1 and E2 were able to significantly increase the numbers of migrating uNK cells, a finding that was unexpected as E1 is considered to be a much ‘weaker’ estrogen than E2. However these assumptions about the activity of E1 are based on results reporting a lower binding affinity for ER and 10-fold lower activation of estrogen response element-driven reporter constructs in cell lines than E2 (Kuiper *et al.*, 1998; Legler *et al.*, 2002). Notably, the E1- and E2-driven increases in uNK cell migration were *both* abrogated by treatment with the pure ER antagonist ICI (Wakeling *et al.*, 1991). The novel finding that E1 increases uNK migration may indicate a role for E1 in maintaining tissue-resident uNK cells during the secretory phase, while increasing concentrations of E2 that occur with decidualisation and establishment of pregnancy (Gibson *et al.*, 2013) may enhance accumulation of uNK cells in decidua and influence recruitment of uNK cells within the endometrium in early pregnancy (Fig. 5; 1 and 2).

Decidualised endometrial stromal cells secrete uNK chemoattractants such as CXCL12 (Kitaya *et al.*, 2005; Wu *et al.*, 2005). The expression and secretion of CXCL12 in endometrial stromal cells is reported to be promoted by E2 but suppressed by progesterone (Glace *et al.*, 2009). Thus, areas of endometrium with increased bioavailability of E2 may have increased concentrations of CXCL12. In the present study, immunofluorescence highlighted the close association of CXCL12-positive stromal cells and CXCR4-positive uNK cells within human decidua and revealed that CXCR4 was immunolocalised to the nuclei of uNK cells (indicative of receptor activation) (Wang *et al.*, 2009). In addition, E2 treatment increased concentrations of CXCR4 mRNA in isolated uNK cells, which suggests that E2 could enhance responsiveness of uNK cells to CXCL12. This may be of increasing importance in early pregnancy as primary human trophoblast cells also secrete CXCL12 (Wu *et al.*, 2005). E2 may therefore influence the recruitment of uNK cells to invading trophoblast cells by altering CXCR4 expression and this may impact on uNK cell activity during the establishment of the materno–fetal interface. Collectively, our new findings provide the first evidence that the bioavailability of estrogens within the decidua could increase uNK cell migration, motility and responsiveness to chemoattractants.

uNK cells promote vascular remodelling during the establishment of pregnancy. The current study investigated the impact of E2 on expression of angiogenic factors in uNK cells. We found that *CCL2* mRNA and protein concentrations were increased by E2 treatment in uNK cells in an ER-dependent manner (Fig. 5; 3 and 4). Transcriptional analysis of uNK cells did not reveal E2-dependent changes in angiogenic factors previously associated with uNK cells such as *VEGFA* and *IFNG*. In addition, *VEGFC* and *PLGF* were not detected by PCR array. This may indicate limited sensitivity of the PCR array or that the secreted proteins are more easily detectable than mRNA transcripts for these genes which may be subject to rapid mRNA turnover. CM from uNK cells contained a number of angiogenic factors all of which may contribute to regulation of the vasculature. We speculate that E2 may modulate production of only a limited subset of angiogenic factors, such as CCL2, which may act in addition to, or in synergism with, other uNK cell-derived factors, such as VEGFA, that are not influenced by the bioavailability of E2. However, blocking E2 action in uNK cells or neutralizing CCL2 abrogated the increases in network formation in our model system consistent with CCL2 being a key target for E2-dependent regulation in uNK cells.

We have previously demonstrated that CCL2 is immunolocalised to perivascular cells in first trimester human decidua (Jones *et al.*, 1997); however, *in vitro* studies suggest that both progestins and E2 inhibit *CCL2* expression in human endometrial stromal cells (Arici *et al.*, 1999). This may indicate that E2-stimulated uNK cells may be the principal source of CCL2 in the endometrial microenvironment in early pregnancy. uNK cells are reported to express chemokine (C-C motif) receptor 2 (CCR2), the CCL2 receptor, and CCL2 is reported to decrease apoptosis and increase proliferation of decidual leukocytes (the majority of which are uNK cells) (He *et al.*, 2012). Thus, CCL2 secreted from uNK cells in response to E2 may have an autocrine impact on uNK cell function and promote persistence/survival in the decidua.

Previous studies investigating the effect of uNK cells on angiogenesis have used human umbilical vein endothelial cells (HUVEC) in network formation assays; however, we and others have reported that endothelial cells from different vascular beds have distinct characteristics and responses (Schatz *et al.*, 2000; Greaves *et al.*, 2013). To the best of our knowledge the results in the current study represent the first *in vitro* evidence of the impact of uNK cells on endothelial cells from human endometrium (HEEC). Interestingly, while E2 is an established regulator of network formation in HUVEC (Morales *et al.*, 1995), recent studies using an *in vitro* model suggest that direct stimulation of human endometrial endothelial cells with E2 has no significant effect on HEEC network formation (Greaves *et al.*, 2013). We speculate that indirect stimulation of the vasculature by E2, relayed via uNK cells, may prevent excessive activation of angiogenesis and lead to controlled, site-specific vascular remodelling in areas of the tissue that have high local bioavailability of E2 and associated accumulation of uNK cells.

Tissue-resident uNK cells are a highly dynamic cell population that varies throughout pregnancy. Gestation-dependent changes in killer-cell immunoglobulin-like (KIR) receptor repertoire have been reported in uNK cells isolated from first trimester decidua from 6 to 12 weeks of gestation (Sharkey *et al.*, 2008; Marlin *et al.*, 2012). This may be particularly important in early pregnancy as changes in NK receptor expression are reported to alter secretion of growth factors and cytokines from uNK cells which in turn may impact on the regulation of endometrial remodelling (Hanna *et al.*, 2006; Sharkey *et al.*, 2008). Consistent with this, functional activity of uNK cells is reported to vary with gestational age. Trophoblast invasion is stimulated by factors secreted from uNK cells from 12 to 14 weeks gestation but not 8–10 weeks gestation and secretion of angiogenic growth factors from uNK cells decreases with gestational age (Lash *et al.*, 2006, 2010). In the present study, uNK cells were isolated from human decidua between 8 and 12 weeks gestation (Supplementary Table SI) which may be a source of variation within our results. Interestingly, despite the variation in gestation the observed responses to E2 were consistent for all end-points which may indicate that E2 regulates uNK cell function independent of gestational age; however, further studies would be required to determine this. Transcriptional profiling suggests that human uNK cells from non-pregnant endometrium are distinct from uNK cells isolated from decidua (Kopcow *et al.*, 2010). In our study we did not assess the impact on uNK cells isolated from non-pregnant endometrium due to the limitations of obtaining a sufficient yield of uNK cells from endometrial pipelle biopsies. However, as decidualisation of stromal cells begins in the mid-secretory phase, further studies are warranted to investigate whether the functional effects of E2 on decidual uNK cells are replicated in uNK cells from non-pregnant endometrium.

In summary, the present study provides mechanistic insight and critical new evidence modelling the physiological regulation of human uNK cell function and cell–cell interactions within the uterine microenvironment. Given our new data, it is conceivable that defects in estrogen-dependent processes may influence uNK cell function and hence targeting estrogen action could provide a new therapeutic strategy.

## Supplementary data

Supplementary data are available at http://humrep.oxfordjournals.org.

## Authors' roles

D.A.G. designed and carried out experimental work and wrote the manuscript. E.G. carried out experimental work. H.O.D.C. designed the work and revised the manuscript. P.T.K.S. designed the work, wrote and revised the manuscript.

## Funding

Studies undertaken in the authors' laboratory were supported by MRC Programme Grant G1100356/1 to P.T.K.S. Funding to pay the Open Access publication charges for this article was provided by MRC programme Grant G1100356/i to P.T.K.S.

## Conflict of interest

None declared.

## Supplementary Material

Supplementary Data
