# Supplementary material for: Estrogen-dependent regulation of human uterine natural killer cells promotes vascular remodelling via secretion of CCL2
Source: Hum Reprod. 2015 Mar 27;30(6):1290–301. doi: 10.1093/humrep/dev067 (PMC4498222; doi:10.1093/humrep/dev067)
Supplement: Supplementary Data [file supp_30_6_1290__index.html]

Estrogen-dependent regulation of human uterine natural killer cells promotes vascular remodelling via secretion of CCL2 — Estrogen-dependent regulation of human uterine natural killer cells promotes vascular remodelling via secretion of CCL2 — Supplementary Data 

# Estrogen-dependent regulation of human uterine natural killer cells promotes vascular remodelling via secretion of CCL2

## Supplementary Data

Supplementary Data

**Files in this Data Supplement:**

- Supplementary data - pdf file
- Supplementary Figure 1 - pdf file
- Supplementary Figure 2 - pdf file
- Supplementary Figure 3 - pdf file
- Supplementary Table 1 - pdf file
- Supplementary Table 2 - pdf file
- Supplementary Table 3 - pdf file
- Supplementary Video 1 - avi file
- Supplementary Video 2 - avi file
