# Supplementary material for: Estrogen-dependent regulation of human uterine natural killer cells promotes vascular remodelling via secretion of CCL2
Source: Hum Reprod. 2015 Mar 27;30(6):1290–301. doi: 10.1093/humrep/dev067 (PMC4498222; doi:10.1093/humrep/dev067)
Supplement: Supplementary Data [file supp_dev067_dev067supp_data.pdf]

Human Reproduction, Vol.0, No.0 2015
doi:10.1093/humrep/dev067

human reproduction

SUPPLEMENTARY DATA

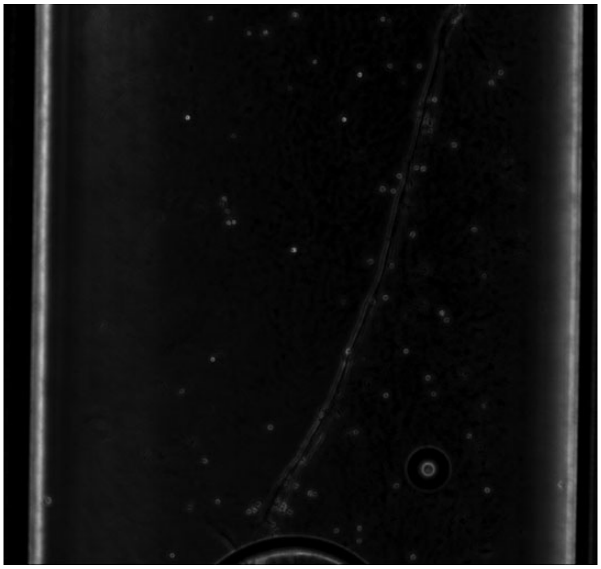

Supplementary Video S1 uNK cell chemokinesis was assessed using time lapse microscopy. Chamber slides were set up containing serum free RPMI 1640 phenol red free media in both reservoirs and uNK cells were treated with vehicle control. Cells were imaged every 5 min for 4 h using Axiovert 200 Inverted Fluorescent Microscope from Zeiss on a temperature controlled stage at 37°C and 5% CO_2. Data were analysed using ImageJ (manual cell tracking plug-in) and chemotaxis and migration tool software (Ibidi). A total of 48 frames were recorded in each treatment, videos are displayed at five frames per second. Analyses are detailed in Fig. 1C and D.

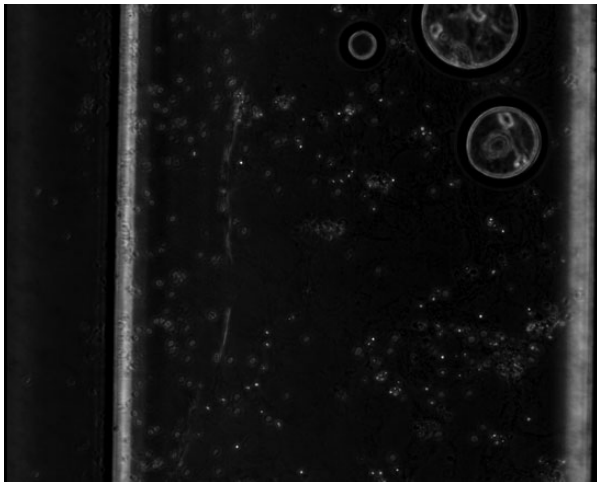

Supplementary Video S2 uNK cell chemokinesis was assessed using time lapse microscopy. Chamber slides were set up containing serum free RPMI 1640 phenol red free media in both reservoirs and uNK cells were treated with E2. Cells were imaged every 5 min for 4 h using Axiovert 200 Inverted Fluorescent Microscope from Zeiss on a temperature controlled stage at 37°C and 5% CO_2. Data were analysed using ImageJ (manual cell tracking plug-in) and chemotaxis and migration tool software (Ibidi). A total of 48 frames were recorded in each treatment; videos are displayed at five frames per second. Analyses are detailed in Fig. 1C and D.
