# Supplementary material for: Estrogen-dependent regulation of human uterine natural killer cells promotes vascular remodelling via secretion of CCL2
Source: Hum Reprod. 2015 Mar 27;30(6):1290–301. doi: 10.1093/humrep/dev067 (PMC4498222; doi:10.1093/humrep/dev067)
Supplement: Supplementary Data [file supp_dev067_dev067supp_fig1.pdf]

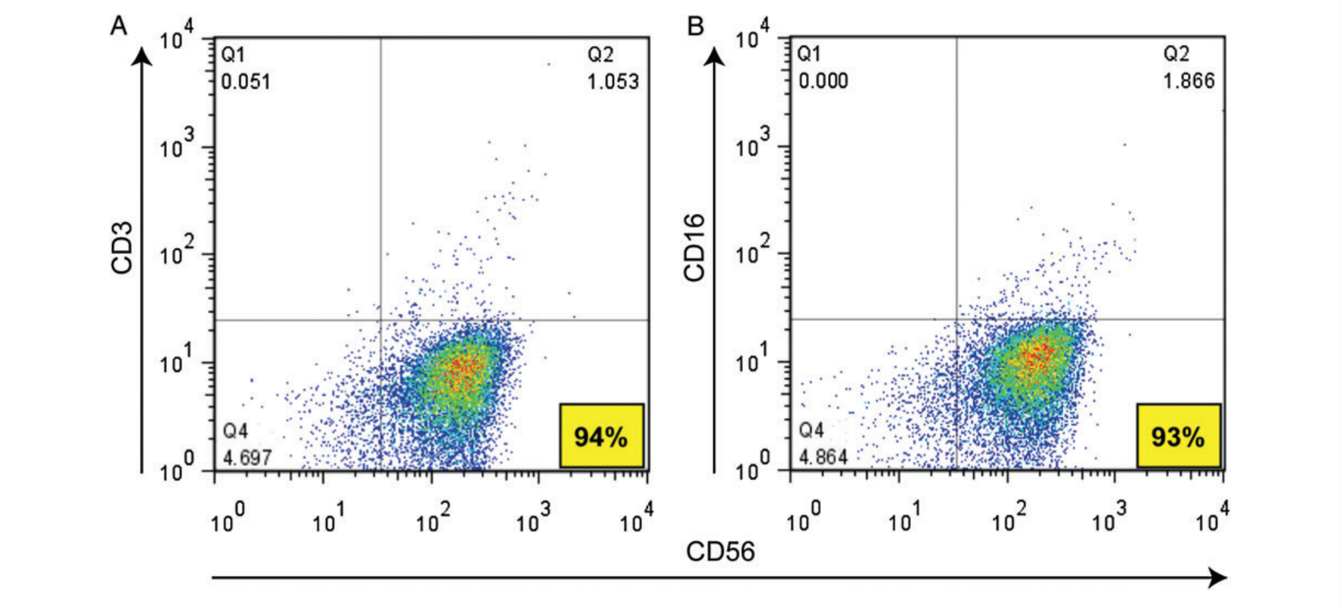

Supplementary Figure S1 Purity of MACS isolated uNK cells was assessed by flow cytometry and determined to be >93% pure and characterised as $CD56^+$ $CD3^-$ $CD16^-$. Viability was confirmed to be >94% by propidium iodide assay (not shown). (**A**) 94% of the total *ungated* cell population was $CD56^+$ and $CD3^-$. (**B**) 93% of the total *ungated* cell population was $CD56^+$ and $CD16^-$.
