# Supplementary material for: Estrogen-dependent regulation of human uterine natural killer cells promotes vascular remodelling via secretion of CCL2
Source: Hum Reprod. 2015 Mar 27;30(6):1290–301. doi: 10.1093/humrep/dev067 (PMC4498222; doi:10.1093/humrep/dev067)
Supplement: Supplementary Data [file supp_dev067_dev067supp_fig2.pdf]

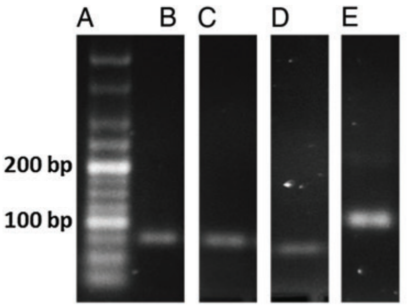

F

	Efficiency (%)	RT negative	NTC	Amplicon size
CCL2	100.83	Undetermined	Undetermined	93
IL15RA	90.62	Undetermined	Undetermined	81
CXCR4	106.06	Undetermined	Undetermined	67
IFNG	97.35	Undetermined	Undetermined	112

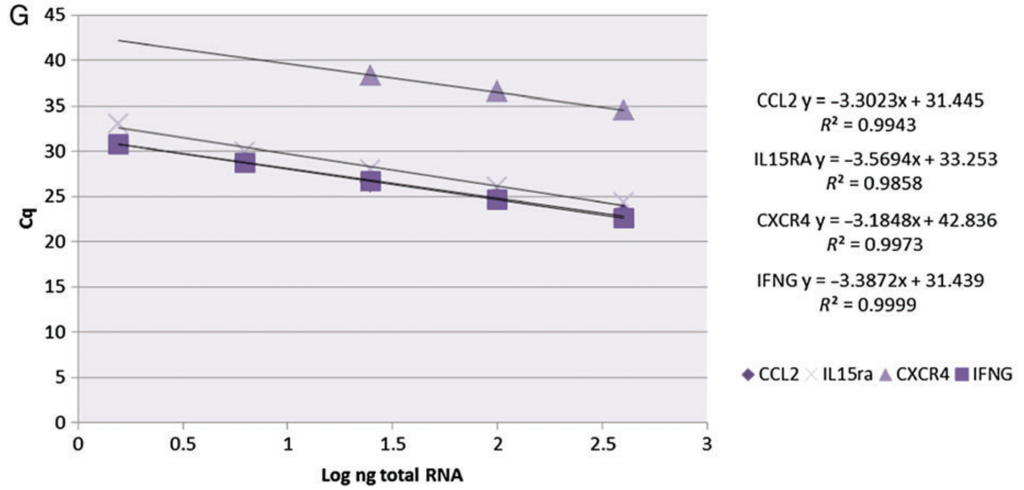

Supplementary Figure S2 Validation of primers used in qPCR experiments; chemokine (C-C motif) ligand 2 (*CCL2*), interleukin 15 receptor alpha (*IL15RA*), chemokine (C-X-C motif) receptor 4 (*CXCR4*) and interferon gamma (*IFNG*). PCR primers were tested by amplifying cDNA synthesised from human uNK cells using Biomix Red PCR kit according to manufacturer's instructions (Bioline). Cycling conditions were as follows; 95°C for 5 min, then up to 35 cycles of 95°C for 30 s, 59°C for 30 s and 72°C for 30 s, followed by 72°C for 10 min. Single bands for PCR target amplicons were detected at the appropriate sizes. (**A**) Ladder (HyperLadder V; Bioline); high intensity bands at 100 and 200 bp: (**B**) *CCL2* (93nt), (**C)** *IL15RA* (81nt), (**D**) *CXCR4* (67nt) and (**E**) *IFNG* (112nt). (**F** and **G**) qPCR primer efficiency for Taqman was assessed by standard curve. Log ng of Total RNA was plotted against Cq (quantification cycle) and the standard curve interpolated; linear standard curve $r^2 > 0.980$ for all primers. PCR efficiency was calculated according to E (efficiency) $= (10^{(-1/\text{slope})} - 1) \times 100$ and was between 90 and 110% for all primers. Non-specific targets were not detected in PCR controls for either reverse transcriptase negative (RT negative) or no template controls (NTC).
