# Supplementary material for: Estrogen-dependent regulation of human uterine natural killer cells promotes vascular remodelling via secretion of CCL2
Source: Hum Reprod. 2015 Mar 27;30(6):1290–301. doi: 10.1093/humrep/dev067 (PMC4498222; doi:10.1093/humrep/dev067)
Supplement: Supplementary Data [file supp_dev067_dev067supp_fig3.pdf]

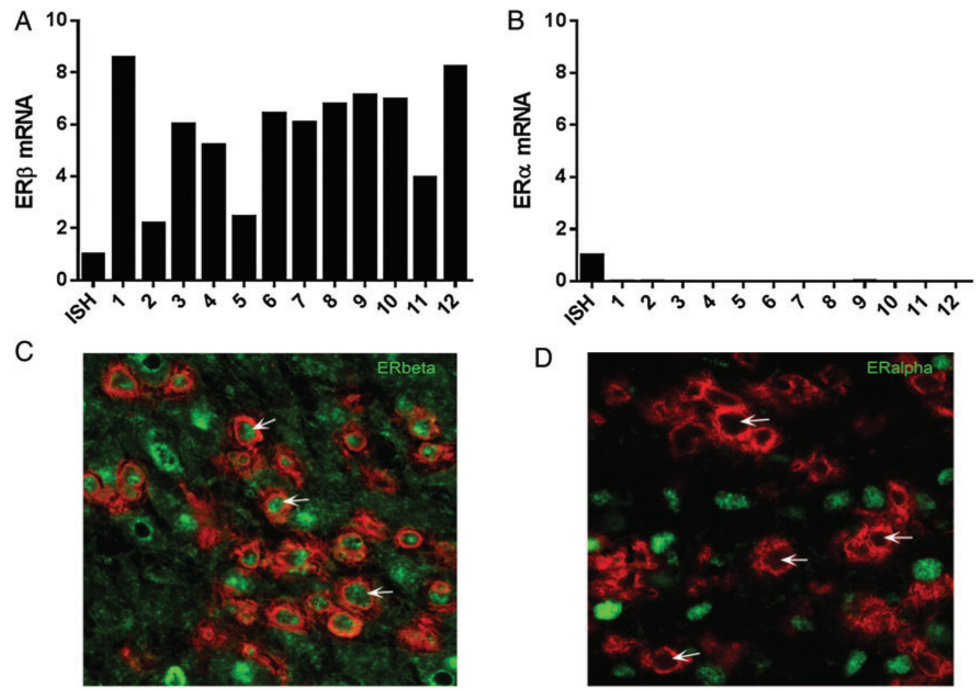

Supplementary Figure S3 The concentration of mRNAs encoding ERβ and ERα was assessed in isolated uNK cells using qPCR. Samples were quantified using the comparative ΔΔCt method relative to ER-positive Ishikawa cells (ISH). (**A**) In freshly isolated primary uNK cells ERβ transcripts were abundant. (**B**) ERα mRNA was low/undetectable in matched samples. Consistent with previous reports (Henderson *et al.*, 2003), immunohistochemistry analysis confirmed *in vivo* ER isoform expression in uNK cells. (**C**) In sections of human first trimester decidua, CD56+ uNK cells (red) stained immunopositive for ERβ (green) in the nucleus (arrows). (**D**) CD56+ uNK cells (red) were immunonegative for ERα and no ERα staining was detected in the nuclei of uNK cells (arrows). Primary antibodies; mouse monoclonal anti-human CD56 (Zymed 18-0152), rabbit polyclonal anti-human ERβ (Santa Cruz biotechnology, sc-8974) and mouse monoclonal anti-human ERα (Vector laboratories, VP-614). Images captured at ×40 magnification.
