# Supplementary material for: Estrogen-dependent regulation of human uterine natural killer cells promotes vascular remodelling via secretion of CCL2
Source: Hum Reprod. 2015 Mar 27;30(6):1290–301. doi: 10.1093/humrep/dev067 (PMC4498222; doi:10.1093/humrep/dev067)
Supplement: Supplementary Data [file supp_dev067_dev067supp_table1.pdf]

Supplementary Table SI **Age of gestation of samples collected for isolation of uNK cells. Average gestation was 10 weeks.**

	Gestation (days)	Gestation (weeks)
Sample 1	56	8.0
Sample 2	56	8.0
Sample 3	56	8.0
Sample 4	57	8.1
Sample 5	58	8.3
Sample 6	59	8.4
Sample 7	60	8.6
Sample 8	62	8.9
Sample 9	63	9.0
Sample 10	68	9.7
Sample 11	69	9.9
Sample 12	70	10.0
Sample 13	71	10.1
Sample 14	71	10.1
Sample 15	71	10.1
Sample 16	72	10.3
Sample 17	72	10.3
Sample 18	72	10.3
Sample 19	73	10.4
Sample 20	73	10.4
Sample 21	74	10.6
Sample 22	74	10.6
Sample 23	75	10.7
Sample 24	75	10.7
Sample 25	75	10.7
Sample 26	76	10.9
Sample 27	77	11.0
Sample 28	78	11.1
Sample 29	79	11.3
Sample 30	79	11.3
Sample 31	79	11.3
Sample 32	81	11.6
Average		**10.0**
