# Supplementary material for: Estrogen-dependent regulation of human uterine natural killer cells promotes vascular remodelling via secretion of CCL2
Source: Hum Reprod. 2015 Mar 27;30(6):1290–301. doi: 10.1093/humrep/dev067 (PMC4498222; doi:10.1093/humrep/dev067)
Supplement: Supplementary Data [file supp_dev067_dev067supp_table2.pdf]

Supplementary Table SII **Conjugated antibodies and isotype controls used in flow cytometry.**

Antibody	Conjugate	Ig	Species	Manufacturer	Product code	Dilution
CD3	RPE	IgG2a	Mouse	AbD Serotec	MCA2184PE	1:50
Isotype	RPE	IgG2a	Mouse	AbD Serotec	MCA929PE	1:50
CD16	RPE	IgG1κ	Mouse	BD Pharmingen	555407	1:50
Isotype	RPE	IgG1κ	Mouse	BD Pharmingen	555749	1:50
CD56	FITC	IgG1κ	Mouse	Abcam	Ab48521	1:20
Isotype	FITC	IgG1κ	Mouse	Abcam	Ab18435	1:20
