# Supplementary material for: Estrogen-dependent regulation of human uterine natural killer cells promotes vascular remodelling via secretion of CCL2
Source: Hum Reprod. 2015 Mar 27;30(6):1290–301. doi: 10.1093/humrep/dev067 (PMC4498222; doi:10.1093/humrep/dev067)
Supplement: Supplementary Data [file supp_dev067_dev067supp_table3.pdf]

Supplementary Table SIII **Table of oligonucleotide sequences used in qPCR analysis.**

Gene name	Forward primer 5′ to 3′	Reverse Primer 5′ to 3′	UPL probe
Homo sapiens chemokine (C-C motif) ligand 2 (CCL2)	ttctgtgcctgctgctcat	ggggcattgattgcatct	83
Homo sapiens CXCR4 (stromal cell derived factor 1 receptor)	ttaagcgcctggtgactgtt	gcccatttcctcggtgtag	47
Homo sapiens interferon, gamma (IFNG)	ggcattttgaagaattggaaag	tttggatgctctggtcatctt	21
Homo sapiens interleukin 15 receptor, alpha (IL15RA)	acaacccccagtctcaaatg	tgccgtcgttactgtggag	37

Reference

Henderson TA, Saunders PT, Moffett-King A, Groome NP, Critchley HO. Steroid receptor expression in uterine natural killer cells. *J Clin Endocrinol Metab* 2003;**88**:440–449.
